# Implementing a Voluntary, Nonregulatory Approach to Nitrogen Management in Tampa Bay, FL: A Public/Private Partnership

**DOI:** 10.1100/tsw.2001.274

**Published:** 2001-11-14

**Authors:** Holly Greening, Bruce D. DeGrove

**Affiliations:** Tampa Bay Estuary Program, St Petersburg, FL 33701, USA

**Keywords:** nutrient management, estuary, voluntary, nonregulatory approach, public/private partnership, Tampa Bay, FL

## Abstract

Participants in the Tampa Bay Estuary Program have agreed to adopt nitrogen-loading targets for Tampa Bay based on the water-quality and related light requirements of underwater seagrasses. Based on modeling results, it appears that light levels can be maintained at necessary levels by “holding the line” at existing nitrogen loadings; however, this goal may be difficult to achieve given the 20% increase in the watershed’s human population and associated 7% increase in nitrogen loading that are projected to occur over the next 20 years. To address the long-term management of nitrogen sources, a nitrogen management consortium of local electric utilities, industries, and agricultural interests, as well as local governments and regulatory agency representatives, has developed a consortium action plan to address the target load reduction needed to “hold the line” at 1992 to 1994 levels. To date, implemented and planned projects collated in the Consortium Action Plan meet and exceed the agreed-upon nitrogen-loading reduction goal. An example of the success of the private partnership aspect of this program can be seen in three phosphate fertilizer mining and manufacturing companies with facilities located on Tampa Bay. These companies are participants in the Estuary Program and the Nitrogen Management Consortium to provide support and input for a program that advocates voluntary, nonregulatory cooperation to reach environmental goals.

## INTRODUCTION

In 1990, Tampa Bay was accepted into the U.S. Environmental Protection Agency's (USEPA's) National Estuary Program. The Tampa Bay National Estuary Program (TBNEP), a partnership that includes three regulatory agencies and six local governments, has built on the resource-based approach initiated by earlier bay-management efforts. Further, it has developed water-quality models to quantify linkages between nitrogen loadings and bay water quality, and models that link loadings and water quality to seagrass goals[1,2,3,4,5,6]. Critical elements of the Tampa Bay process are

1. To set specific, quantitative seagrass coverage goals for each bay segment;
2. To determine seagrass water-quality requirements and appropriate nitrogen-loading targets; and
3. To define and implement the nitrogen-management strategies needed to achieve the load-management targets.

## STEP 1. SET QUANTITATIVE RESOURCE MANAGEMENT GOALS

The establishment of clearly defined and measurable goals is crucial for a successful resource-management effort. The TBNEP Management Conference adopted the initial goal to increase the current Tampa Bay seagrass cover to 95% of that present in 1950[7].

Based on digitized aerial photographic images, it was estimated that approximately 16,500 ha of seagrass existed in Tampa Bay in 1950[8]. At that time, seagrasses grew to depths of 1.5 to 2 m in most areas of the bay. By 1992, approximately 10,400 ha of seagrass remained in Tampa Bay, a loss of more than 35% since the 1950 benchmark period. Some of the observed loss (about 160 ha) occurred as the result of direct habitat destruction associated with the construction of navigation channels and other dredging and filling projects within existing seagrass meadows and is assumed to be nonrestorable through water-quality management actions.

In 1996, the TBNEP adopted a bay-wide minimum seagrass goal of 15,400 ha. This goal represented 95% of the estimated 1950 seagrass cover (minus the nonrestorable areas) and includes the protection of the existing 10,400 ha and the restoration of an additional 5000 ha[7].

## STEP 2. DETERMINE SEAGRASS WATER-QUALITY REQUIREMENTS AND APPROPRIATE NITROGEN-LOADING RATES

Once the seagrass restoration and protection goal was established by the participants, the next steps established the environmental requirements necessary to meet the agreed-upon goal and subsequent management actions necessary to meet those requirements[6].

### A. Determine Environmental Requirements Needed to Meet the Seagrass Restoration Goal

Recent research indicates that the deep edges of *Thalassia testudinum* meadows, the primary seagrass species for which nitrogen-loading targets are being set, correspond to the depth at which 20.5% of subsurface irradiance (the light that penetrates the water surface) reaches the bay bottom on an annual average basis[9]. The long-term seagrass coverage goal can thus be restated as a water clarity and light-penetration target. Therefore, to restore seagrass to near 1950 levels in a given bay segment, water clarity in that segment should be restored to the point that allows 20.5% of subsurface irradiance to reach the same depths that were reached in 1950.

### B. Determine Water Clarity Necessary to Allow Adequate Light to Penetrate to the 1950 Seagrass Depths

Water clarity and light penetration in Tampa Bay are affected by a number of factors, such as phytoplankton biomass, nonphytoplankton turbidity, and water color. Janicki and Wade[2] used regression analyses based on long-term data from Tampa Bay to develop an empirical model describing water clarity variations in the four largest bay segments (Old Tampa Bay, Hillsborough Bay, Middle Tampa Bay, and Lower Tampa Bay) (Fig. 1).

Water color may be an important cause of light attenuation in some bay segments; however, including color in the regression model did not produce a significant improvement in the predictive ability of the model. Results of the modeling effort indicate that, on a bay-wide basis, variation in chlorophyll *a* concentration is the major factor affecting variation in average annual water clarity.

### C. Determine Chlorophyll *a* Concentration Targets Necessary to Maintain Water Clarity Needed to Meet the Seagrass Light Requirement

The empirical regression model was used to estimate chlorophyll *a* concentrations necessary to maintain water clarity needed for seagrass growth for each major bay segment. The adopted segment-specific annual average chlorophyll *a* targets (8.5 $\mu$g/l for Old Tampa Bay, 13.2 $\mu$g/l for Hillsborough Bay, 7.4 $\mu$g/l for Middle Tampa Bay, and 4.6 $\mu$g/l for Lower Tampa Bay) are easily measured and tracked through time, and are used as intermediate measures for assessing success in maintaining water-quality requirements necessary to meet the long-term seagrass goal.

### D. Determine Nutrient Loadings Necessary to Achieve and Maintain the Chlorophyll *a* Targets

Water-quality conditions in the 1992 to 1994 period appeared to allow an annual average of more than 20.5% of subsurface irradiance to reach target depths (i.e., the depths to which seagrasses grew in 1950) in three of the four largest bay segments (Hillsborough Bay, Old Tampa Bay, and Lower Tampa Bay). Water quality in the Middle Tampa Bay segment currently allows slightly less than 20.5% to target depth; thus, a management strategy based on “holding the line” at the 1992 to 1994 nitrogen-loading rates should be adequate to achieve the seagrass restoration goals in three of the four segments. This “hold the line” approach, combined with careful monitoring of water quality and seagrass extent, was adopted by the TBNEP partnership in 1996 as its initial nitrogen-load management strategy.

As an additional complicating factor, a successful adherence to the “hold the line” nitrogen-loading strategy may be hindered by the projected population growth in the watershed. A 20% in-

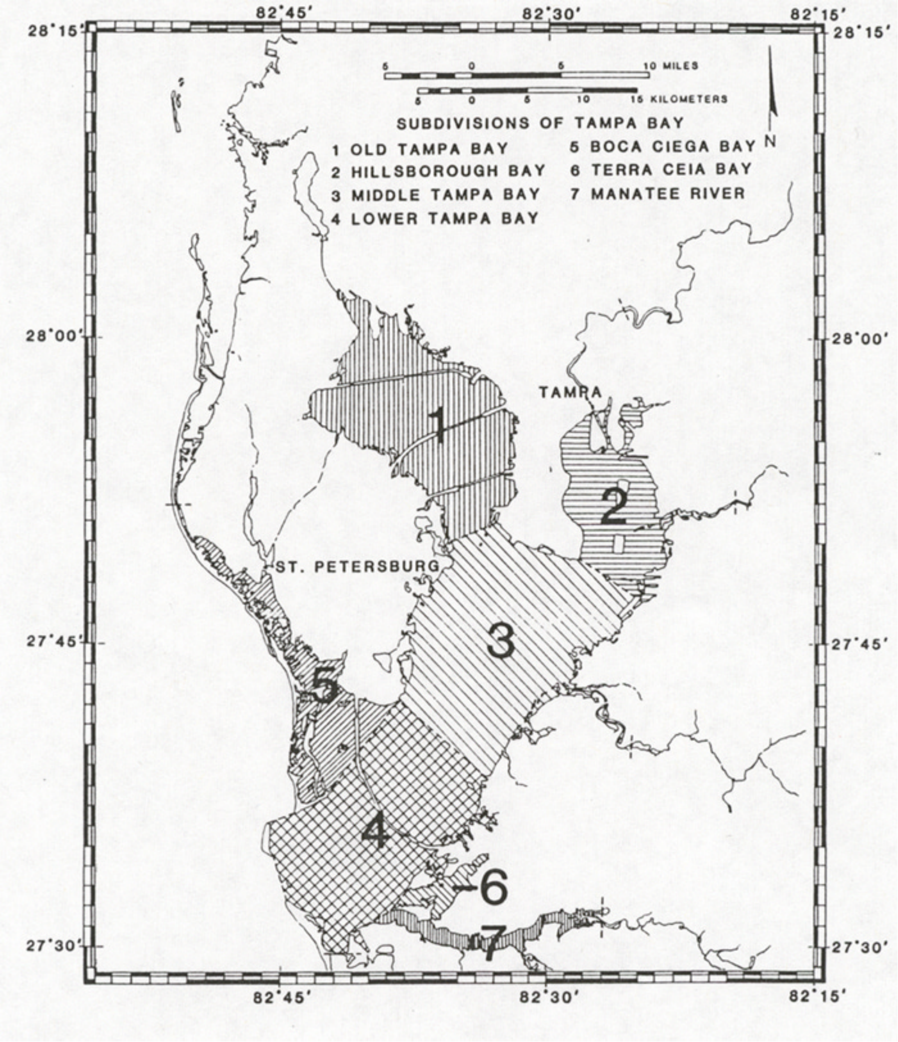

**FIGURE 1.** Major bay segments of the Tampa Bay estuary.

crease in population and a 7% increase in annual nitrogen load are anticipated by the year 2010[10]. Therefore, if the projected loading increase (a total of 17 U.S. tons/year) is not prevented or precluded by watershed-management actions, the "hold the line" load-management strategy will not be achieved.

## STEP 3: DEFINE AND IMPLEMENT NITROGEN-MANAGEMENT STRATEGIES NEEDED TO ACHIEVE LOAD-MANAGEMENT GOALS

Local government and agency partners in the TBNEP signed an intergovernmental agreement in 1998, pledging to carry out specific actions needed to "hold the line" on nitrogen loadings. The Intergovernmental Agreement includes the responsibility of each partner for meeting the nitrogen-management goals and a timetable for achieving them. How those goals are reached will be left up to the individual communities as defined by them in their action plans[11]. The Tampa Bay National Estuary Program was also renamed the Tampa Bay Estuary Program (TBEP) as part of progression from the planning phase to implementation of the adopted Comprehensive Conservation and Management Plan.

To maintain nitrogen loadings at 1992 to 1994 levels, local government action plans address reductions of that portion of the nitrogen target which relates to nonagricultural stormwater runoff and municipal point sources within their jurisdictions. To address reductions attributed to atmospheric deposition, industrial and agricultural sources, and springs, a nitrogen management consortium of local electric utilities, industries, and agricultural interests, as well as the local governments and regulatory agency representatives in the TBEP, was established (Table 1). Table 2 shows the cumulative nitrogen load-reduction targets adopted by each participating jurisdiction and the consortium for 1995 to 1999 for each bay segment. The Nitrogen Management Action Plan developed by the public and private partners in the consortium combines for each bay segment all local government, agency, and industry projects that will contribute to meeting the 5-year nitrogen-management goal[12].

The types of nutrient-reduction projects included in the Consortium Action Plan range from traditional nutrient-reduction projects such as stormwater upgrades, industrial retrofits, and agricultural best-management practices to actions not primarily associated with nutrient reduction, such as land acquisition and habitat restoration projects. A total of 105 projects submitted by local governments, agencies, and industries are included in the plan; 95% of these projects address nonpoint sources and

**TABLE 1**
**Public and Private Partners of the Tampa Bay Nitrogen Management Consortium, July 2001**

**Public Partners**

- City of Clearwater
- City of St. Petersburg
- City of Tampa
- Environmental Protection Commission of Hillsborough County
- Florida Department of Agriculture and Consumer Services
- Florida Department of Environmental Protection
- Florida Fish and Wildlife Commission/Florida Marine Research Institute
- Hillsborough County
- Manatee County
- Manatee County Agricultural Extension Service
- Pinellas County
- Southwest Florida Water Management District
- Tampa Bay Estuary Program
- Tampa Bay Regional Planning Council
- Tampa Port Authority
- U.S. Army Corps of Engineers
- U.S. Environmental Protection Agency

**Private Partners**

- Cargill Fertilizer, Inc.
- CF Industries, Inc.
- CSX Transportation
- Eastern Associated Terminals Company
- Florida Phosphate Council
- Florida Power & Light
- Florida Strawberry Growers Association
- IMC-Phosphates Company
- Pakhoed Dry Bulk Terminals
- Tampa Electric Company

**TABLE 2**
**Tampa Bay Nitrogen Reduction Goals By Jurisdiction and for the Tampa Bay Nitrogen Management Consortium (Total Nitrogen in U.S. Tons)**

| | CUMULATIVE 1995–1999 GOALS FOR NITROGEN REDUCTION/MANAGEMENT | | | | | | | Total (reduction in annual load) (tons) |
|---|---|---|---|---|---|---|---|---|
| Source Category | Pinellas County | Clearwater | St. Petersburg | Hillsborough County | Tampa | Manatee County | TBEP Consortium | |
| Old Tampa Bay | 0.30 | 0.20 | 0.05 | 0.40 | 0.10 | <0.01 | 1.05 | 2.10 |
| Hillsborough Bay | <0.01 | <0.01 | <0.01 | 4.75 | 8.45 | <0.01 | 28.25 | 41.50 |
| Middle Tampa Bay | <0.01 | <0.01 | 0.90 | 2.50 | <0.01 | 0.50 | 7.15 | 11.05 |
| Lower Tampa Bay | <0.01 | <0.01 | <0.01 | <0.01 | <0.01 | 8.35 | 17.00 | 25.35 |
| Boca Ciega Bay | 0.85 | <0.01 | 1.05 | <0.01 | <0.01 | <0.01 | 2.00 | 3.90 |
| TOTAL | 1.15 | 0.20 | 2.00 | 7.65 | 8.55 | 8.85 | 55.45 | 83.85 |
| % | 1.4 | 0.2 | 2.4 | 9.1 | 10.2 | 10.6 | 66.1 | 100.0 |

*Note*: TBEP = Tampa Bay Estuary Program

**TABLE 3**
**Tampa Bay Nitrogen Management Consortium Summary of Goals and Expected Reductions (Cumulative tons TN reduced or precluded/year by 2000)**

| Bay Segment | 1995–1999 Nitrogen Reduction Goal | Expected Reduction: Completed or Ongoing Projects[1] | Expected Reduction: Pending Projects[2] | Expected Reduction: Atmospheric Deposition[3] |
|---|---|---|---|---|
| Old Tampa Bay | 2.1 | 3.6 | 1.5 | 3.6 – 6.2 |
| Hillsborough Bay | 41.5 | 62.0 | 3.9 | 13.9 – 24.0 |
| Middle Tampa Bay | 11.1 | 14.8 | 6.3 | 4.6 – 7.9 |
| Lower Tampa Bay | 25.4 | 25.0 | 11.2 | 5.7 – 10.0 |
| Boca Ciega Bay | 3.9 | 0.8 | 4.8 | 1.2 – 2.1 |
| **Total** | 84.0 | 106.2 | 27.7 | 29.0 – 50.2 |

[1] Projects have been completed or were under construction (2000). These summaries do not include reductions expected from Tampa Electric Company (TECO) atmospheric deposition reductions.
[2] Projects have funding available and were in the planning or permitting stages (2000). These summaries do not include reductions expected from TECO atmospheric deposition reductions.
[3] Range of atmospheric deposition reductions expected from TECO, as estimated by the Environmental Protection Commission of Hillsborough County (first estimate) and TECO (second estimate).

account for 71% of the expected total nitrogen reduction. Half (50%) of the total load reduction will be achieved through public sector projects and 50% by industry.

Table 3 summarizes expected reductions from those projects that were completed by the end of 1999, and represent both reduction in existing loads and preclusion of expected loads if projects had not been implemented. A total reduction in nitrogen loading to Tampa Bay of 134 U.S. tons/year (expressed as total nitrogen) was expected by the end of 2000 as a result of action plan projects; this reduction exceeds by 60% the 1995 to 1999 reduction goal of 84 tons/year. An updated estimate of nitrogen loadings to the bay from all sources was initiated by TBEP in 2001, after which the effectiveness of the proposed projects in maintaining loads to the bay will be evaluated.

The approach advocated by the TBEP stresses cooperative solutions and flexible strategies to meet nitrogen-management goals. This approach does not prescribe the specific types of projects that must be included in the Action Plan; consortium partners have been encouraged to pursue the most cost-effective options to achieve the agreed-upon goals for nitrogen management.

The TBEP will review and revise nitrogen-management goals every 5 years, or more often if significant new information becomes available. If it is found that nitrogen-reduction goals or chlorophyll targets are not being met at some point in the future, the consortium has agreed to assess and implement alternative methods — including nitrogen-loading trading and large collaborative projects — as potential methods for reaching goals.

One example of the cooperative partnership approach advocated by the TBEP can be seen in the participation of the phosphate industry. The phosphate industry, with several facilities located on the bay and other facilities within the watershed, is a very visible source of loadings to Tampa Bay. The industry, in deciding to participate in the Estuary Program and the Nitrogen Management Consortium, determined that participation provides benefits to the industry, the Estuary Program, and the surrounding community. Participation in these programs allows the industry to be part of the decision-making process and to provide others with information about the phosphate industry as it operates today. The phosphate industry is committed to making facility improvements that reduce loadings to the bay. Participation in the Estuary Program provides an opportunity to receive credit in the public arena for these improvements. The voluntary nature of these programs has encouraged flexibility, innovation, and a cooperative attitude among participants, providing benefits to the bay while avoiding mandatory regulatory requirements that can deter the desire to go beyond minimum requirements.

## CONCLUSIONS

The Tampa Bay management community has agreed that the protection and restoration of Tampa Bay's living resources is of primary importance and have, through the TBEP process (initiated in 1991), adopted nitrogen-loading targets for Tampa Bay based on the water-quality requirements of *Thalassia testudinum* and other native seagrass species. A long-term goal has been adopted to achieve 15,400 ha of seagrass in Tampa Bay, or 95% of that observed in 1950. To reach the long-term seagrass restoration goal, a 7% increase in nitrogen loading associated with a projected 20% increase in the watershed's human population over the next 20 years must be offset. Government and agency partners in the TBEP and private industries and interests participating in the Nitrogen Management Consortium have identified and committed to specific nitrogen-load reduction projects to ensure that the water quality conditions necessary to meet the long-term living resource restoration goals for Tampa Bay are achieved.

The opportunity for private industry to participate in a voluntary management program has resulted in concrete actions by industry to reduce loadings to the bay, reductions that were unlikely to occur in a "command and control" regulatory environment more typically expected in Florida. The result to date has been an improved environment with less regulatory effort and a cooperative attitude among public and private entities that holds promise far into the future. The Tampa Bay nitrogen management example demonstrates that voluntary public/private partnerships can be an effective approach to solving complex and costly watershed management issues.

| | | |
|---|---|---|
| Received: | July | 18, 2001 |
| Revised: | September | 18, 2001 |
| Accepted: | October | 5, 2001 |
| Published: | November | 14, 2001 |

## BIOSKETCH

**Holly Greening**, Senior Scientist for the Tampa Bay Estuary Program, has 20 years experience in marine and estuarine ecology and management. Her primary research interests since joining EPA's National Estuary Program system in Tampa Bay in 1991 include estuarine eutrophication sources and management, atmospheric deposition, and watershed management. Linking science and management to address natural resource restoration goals using multi-partner approaches (including public and private entities) has been a major focus of her work in Tampa Bay.

**Bruce DeGrove**, Director of Regulatory Affairs for the Florida Phosphate Council, served 19 years with Florida's environmental regulatory agency in water programs before joining the Florida Phosphate Council in 1993. He is currently responsible for addressing surface water, groundwater, land reclamation, and air emissions issues for the phosphate mining and phosphate chemical processing industry.
